# Supplementary material for: Developing an Advanced PM2.5 Exposure Model in Lima, Peru
Source: Remote Sens (Basel). Author manuscript; Available in PMC 2019 Aug 1. (PMC6671674; doi:10.3390/rs11060641)
Supplement: Supplemental [file NIHMS1033584-supplement-Supplemental.pdf]

Supplemental Material

Developing advanced $PM_{2.5}$ exposure models in Lima, Peru

Bryan N Vu[a], Odón Sánchez[b], Jianzhao Bi[a], Qingyang Xiao[a], Nadia N Hansel[c], William Checkley[c], Kyle Steenland[a], Yang Liu[a*]

[a] Department of Environmental Health, Rollins School of Public Health, Emory University, Atlanta, GA, United States.

[b] Carrera Profesional de Ingeniería Ambiental, Universidad Nacional Tecnológica de Lima Sur (UNTELS), Av. Central, Villa El Salvador, Lima, Peru.

[c] Division of Pulmonary and Critical Care, School of Medicine, Johns Hopkins University, Baltimore, MD, United States.

[*]**Address for correspondence:**

Yang Liu, PhD

Department of Environmental Health, Rollins School of Public Health, Emory University, Atlanta, GA 30322, USA

1518 Clifton Road NE, Atlanta, GA 30322, USA

Email: yang.liu@emory.edu

Number of pages: 5

1. Supplemental Methods

Measurements for daily average temperature and relative humidity from Weather Underground were correlated with temperature from WRF-Chem and relative humidity from ECMWF. In Figure S1, we show the simple correlation matrix between these variables. Correlation coefficients between Weather Underground temperature with WRF-Chem temperature and ECMWF relative humidity were 0.69 and 0.59, respectively. Correlation coefficients between Weather Underground relative humidity with WRF-Chem temperature and ECMWF relative humidity were both 0.05, respectively. The correlation coefficient between Weather Underground temperature and relative humidity was 0.17.

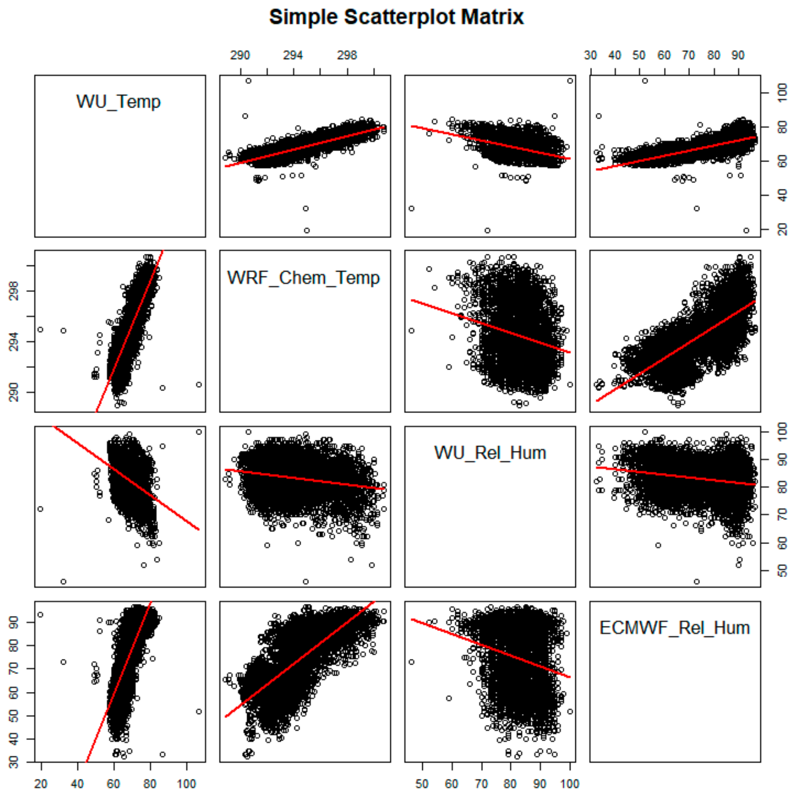

Figure S1. Simple Correlation Matrix between Weather Underground temperature and relative humidity with WRF-Chem temperature and ECMWF relative humidity.

2. Supplemental Results

Histograms of all the predictors used in the modeling approach can be seen in Figure S2. Variables including AOD, surface solar radiation downwards (SSRD, or the solar radiation in the downward direction at the surface), NDVI, temperature, PBL, and wind U component were considered normally distributed. Distance from monitor to nearest major road, elevation, percent urbanization, and population are assumed to be mostly static between years and non-normally distributed due to limited number of ground monitors with 6 of the 10 monitors (JHU sites) densely clustered within a region. WRF-Chem $PM_{2.5}$ was right skewed while wind V component was left skewed. WRF-Chem albedo, cloud fraction, and pressure are also non-normally distribution, which may also be a result of the location and distribution of the monitor stations.

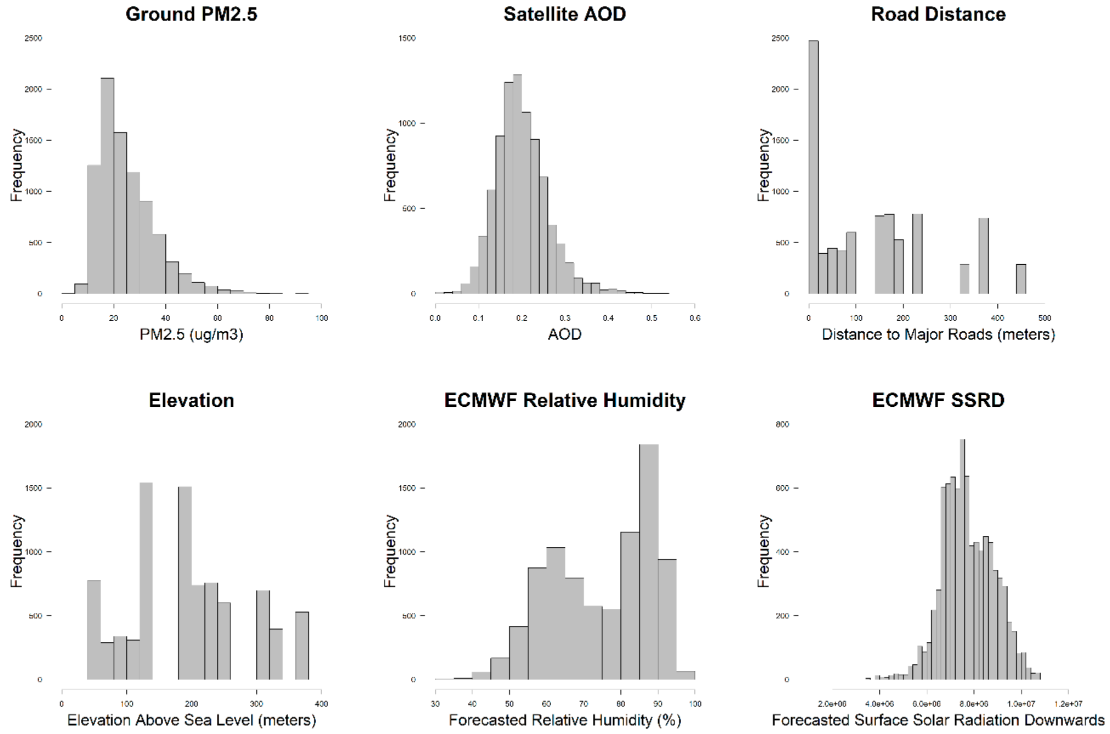

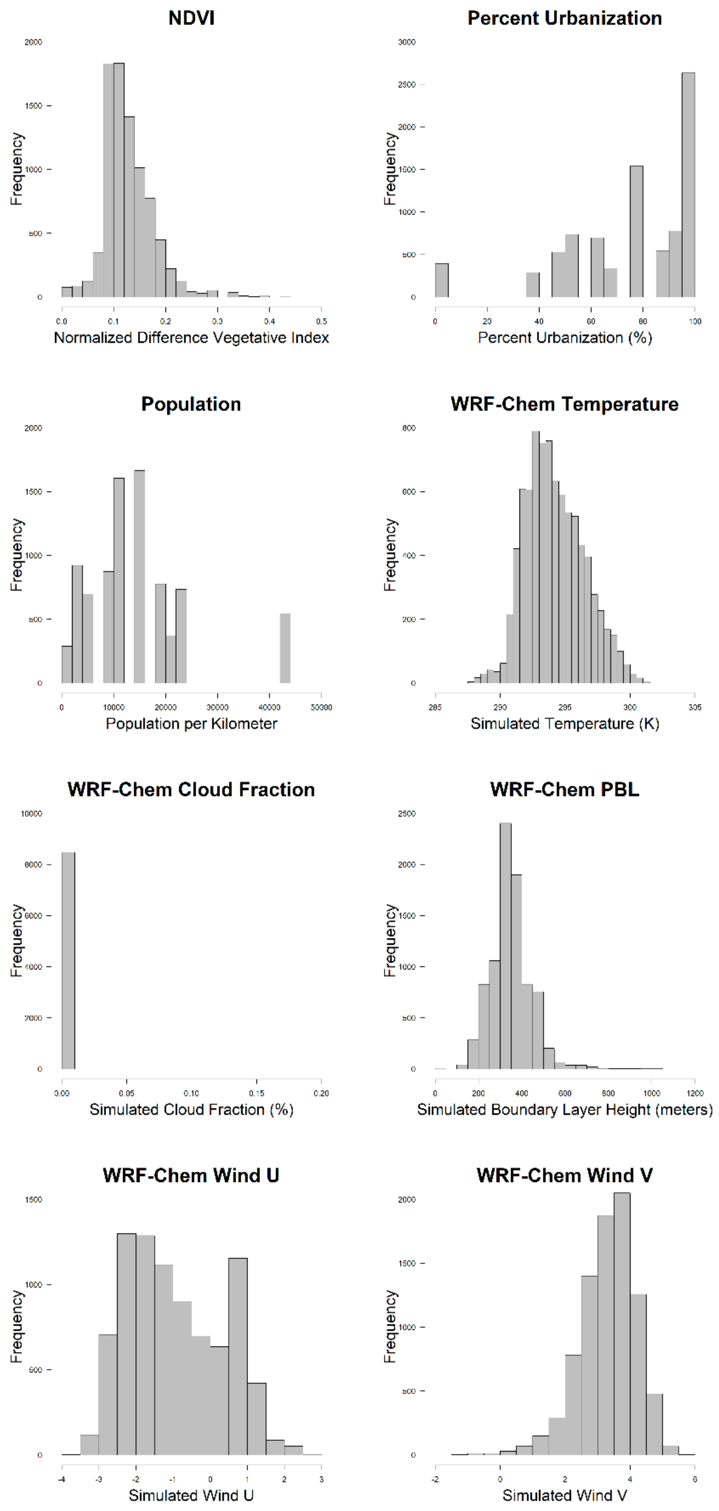

Figure S2. Histograms of each predictor variable.

In Figure S3, we show the monthly mean prediction maps of $PM_{2.5}$ in μg/m^3 for 2015. Overall, concentrations of $PM_{2.5}$ spatially increases from the month of April and peaks during June before decreasing to December, aggreeing with the monthly mean estimates from the ground monitors.

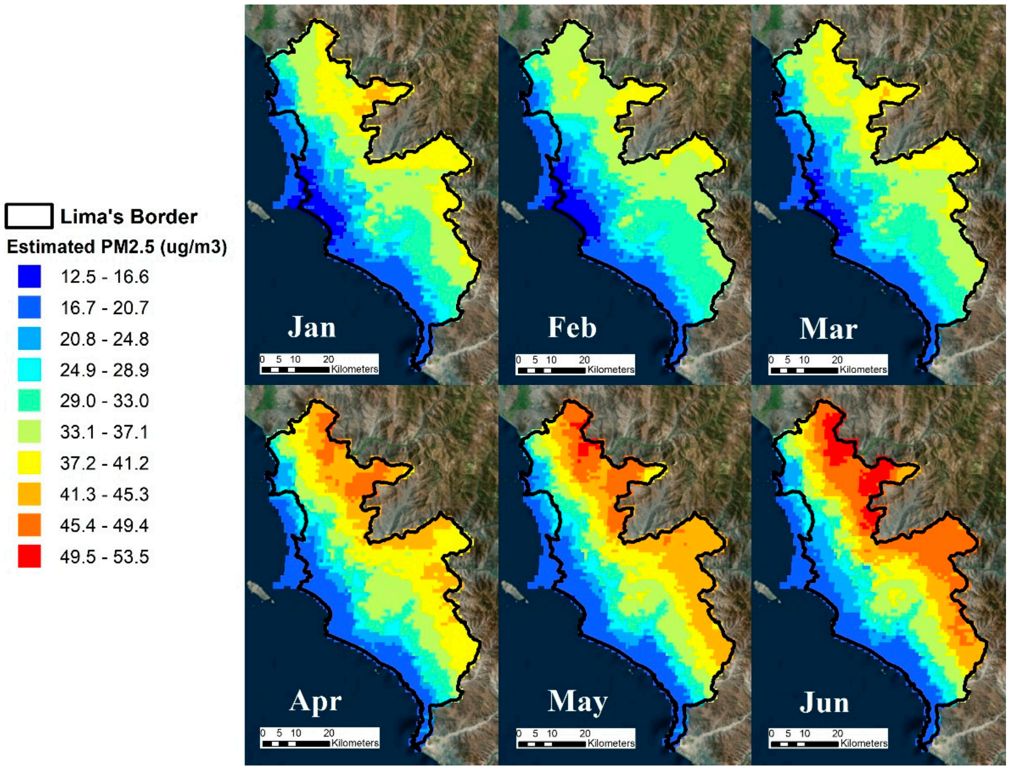

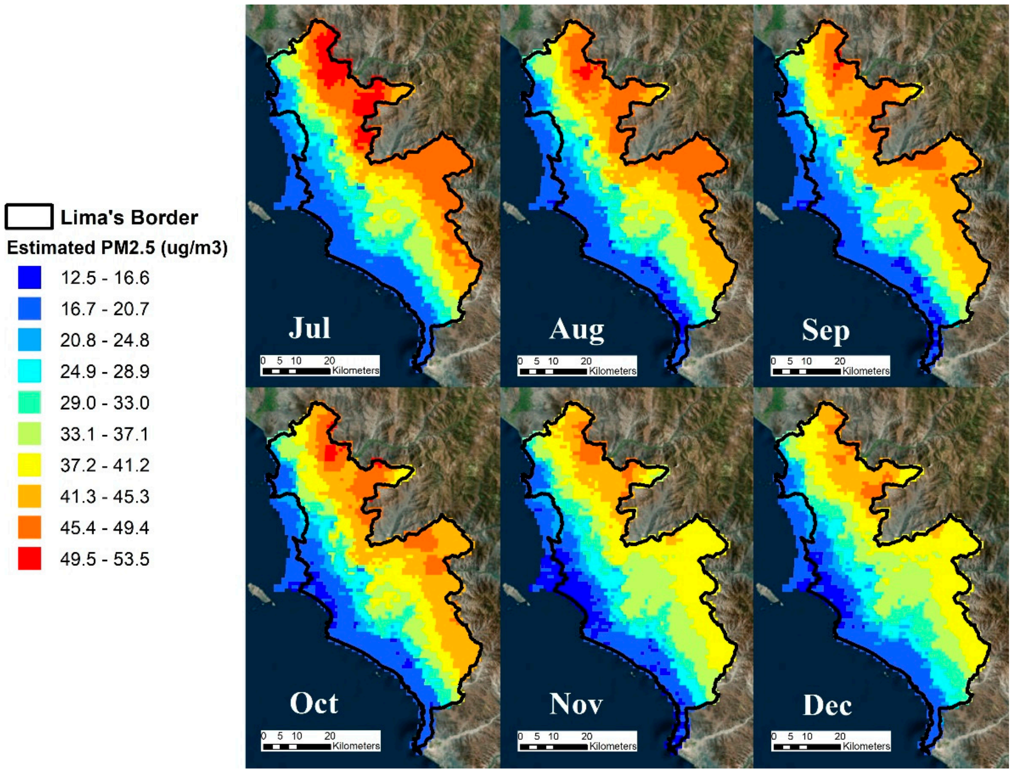

Figure S3. Monthly mean prediction maps of $PM_{2.5}$ concentrations in μg/m^3 for 2015.
